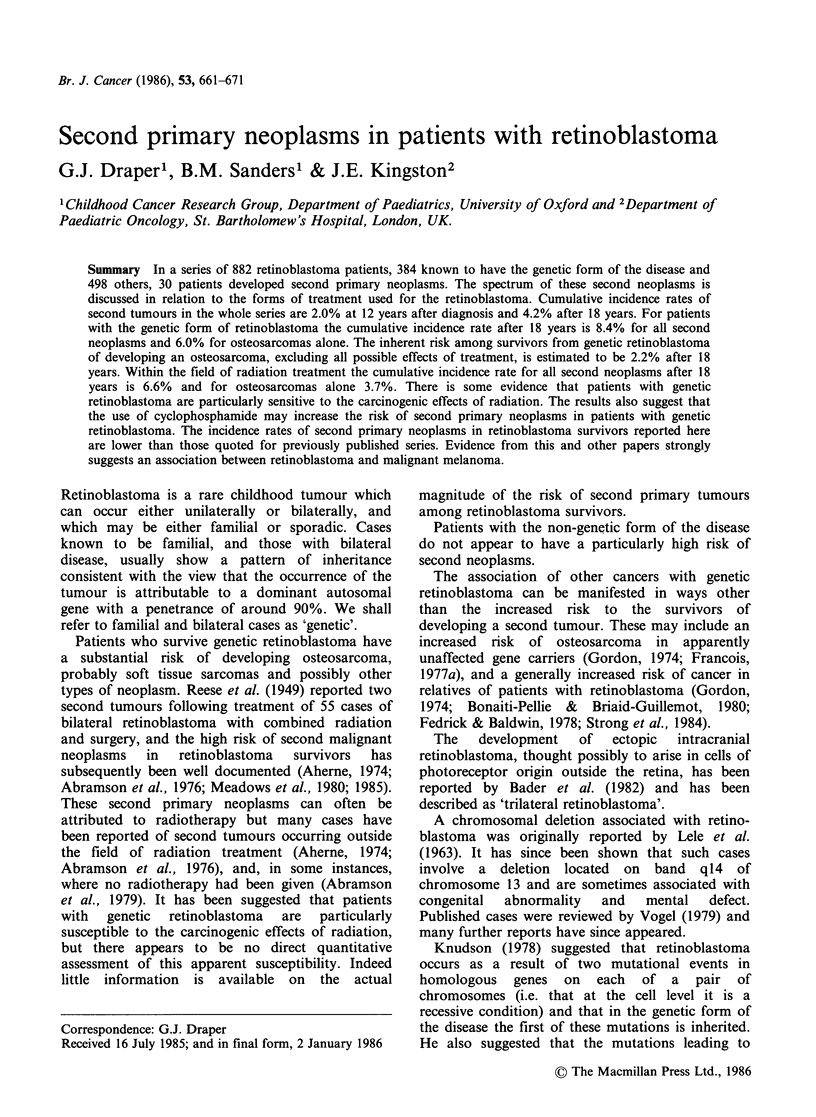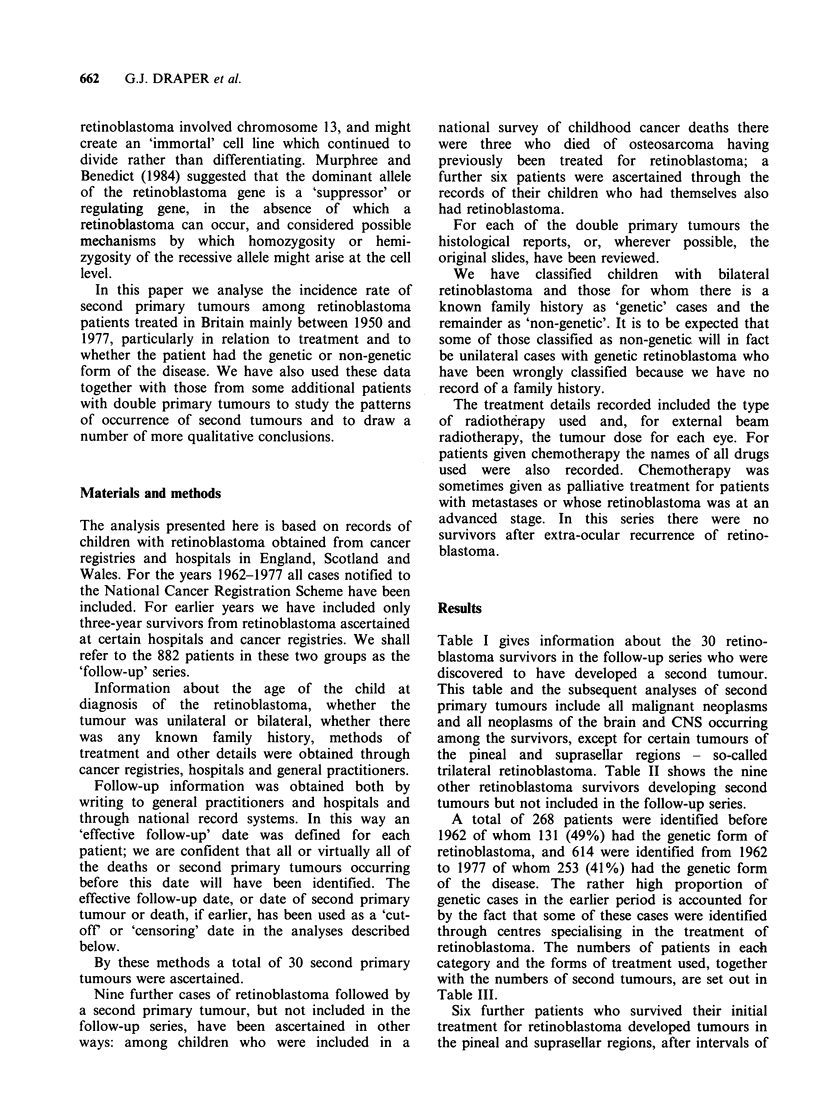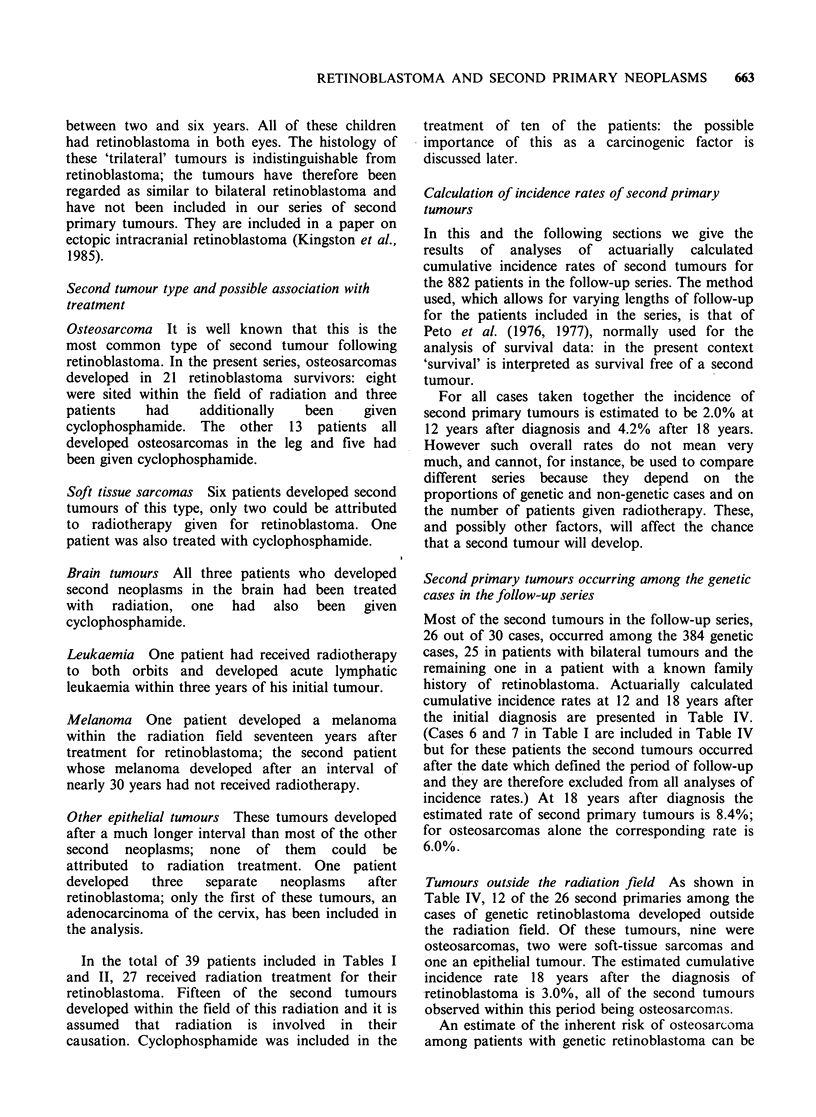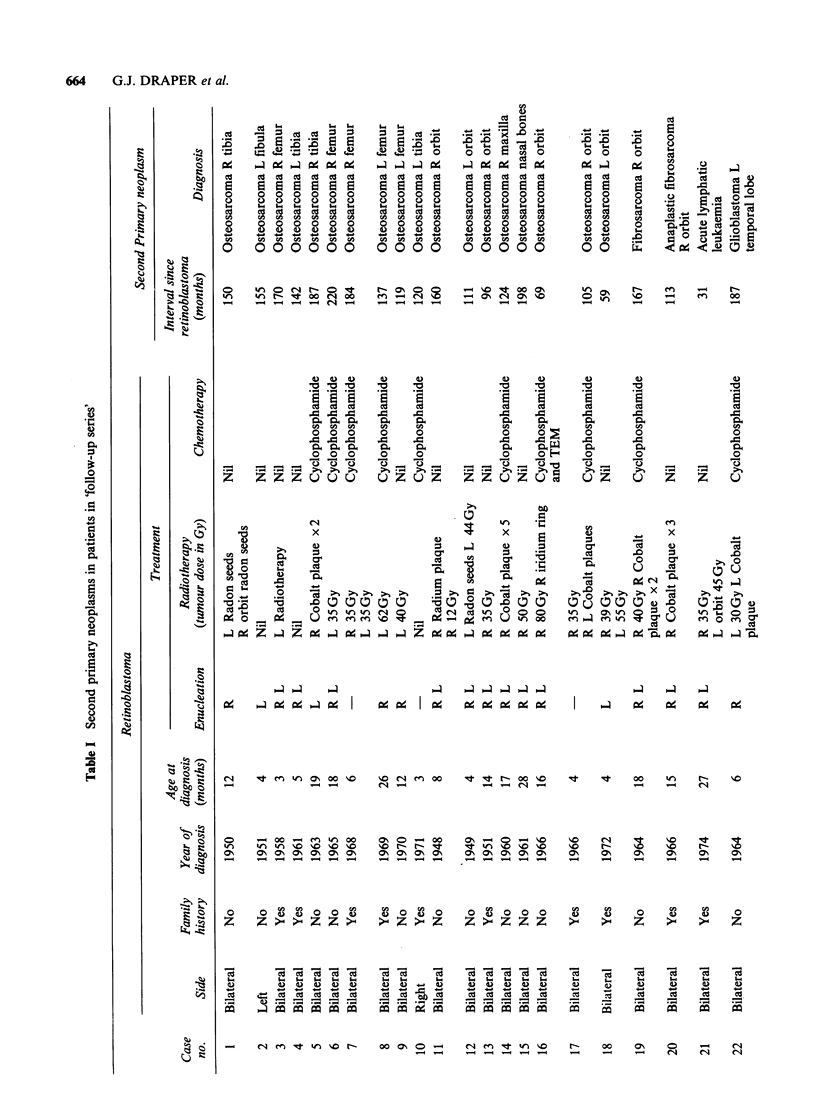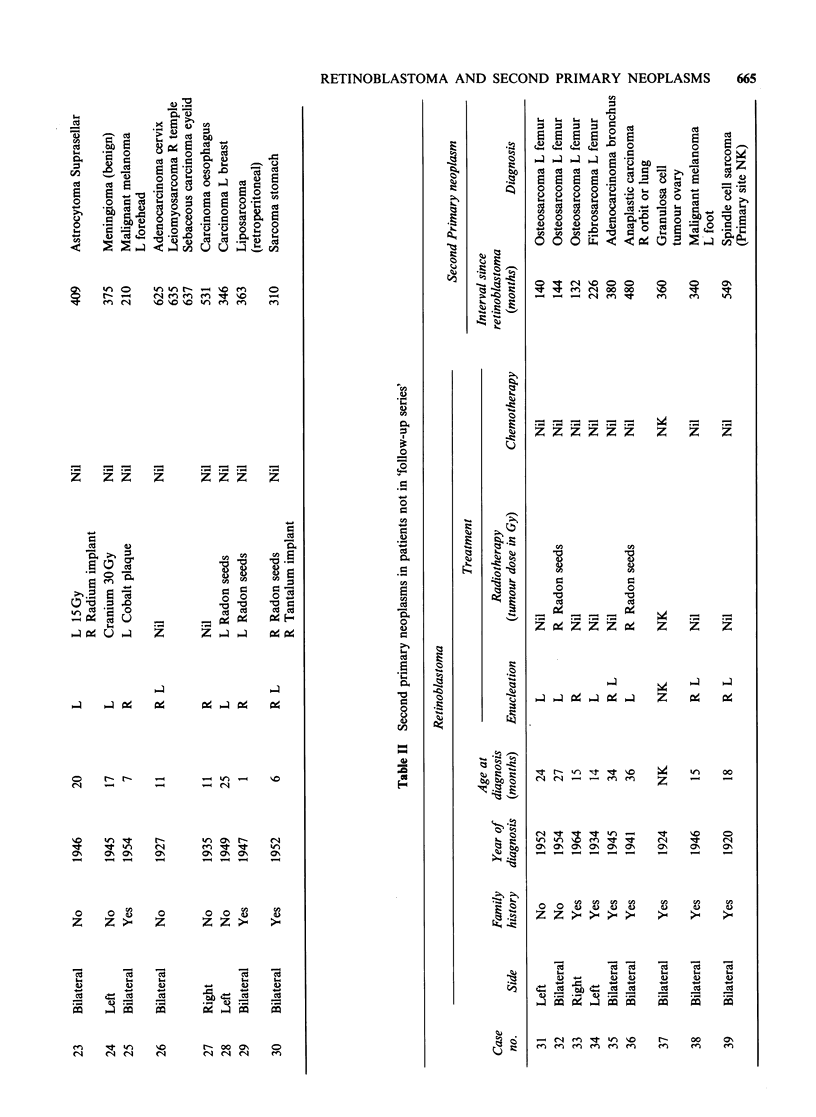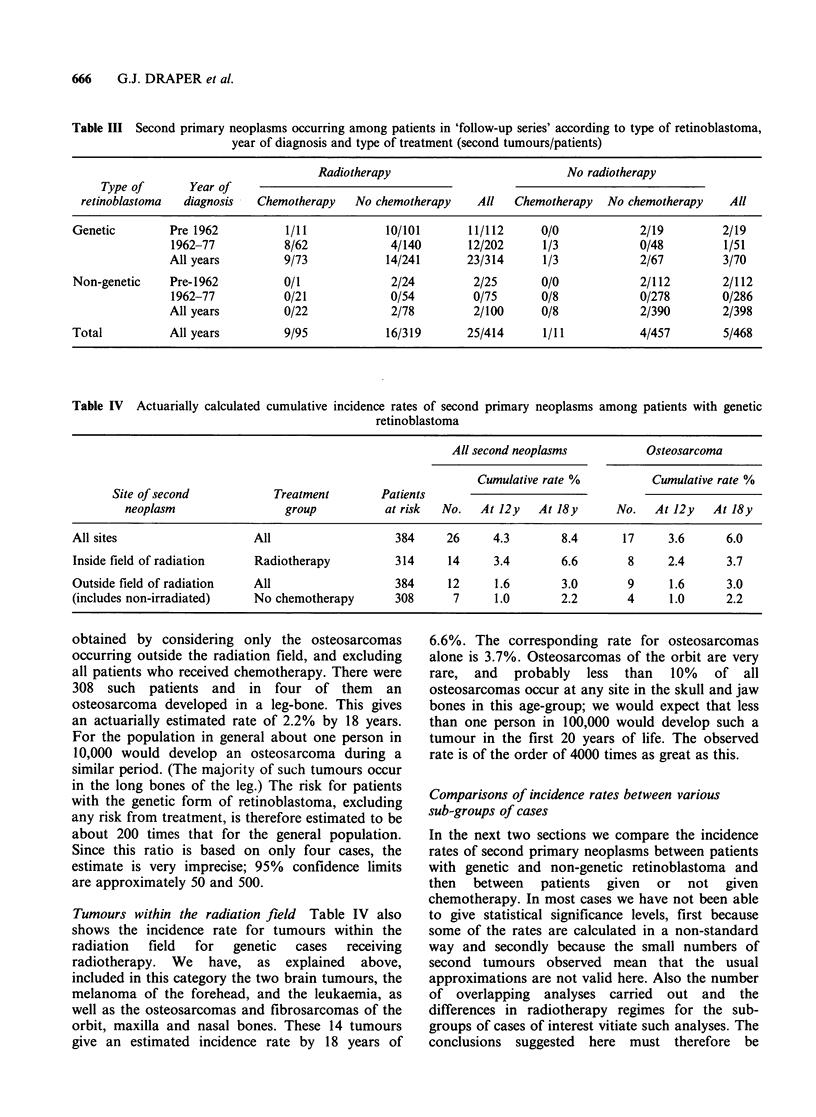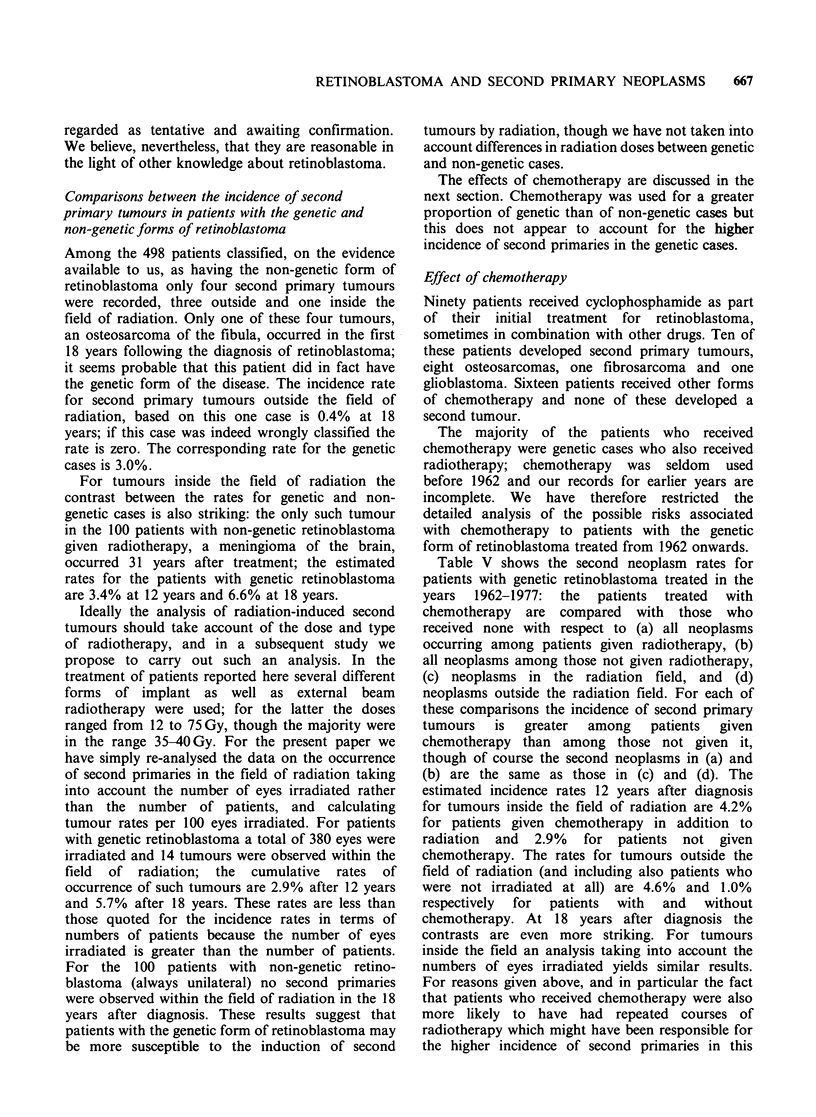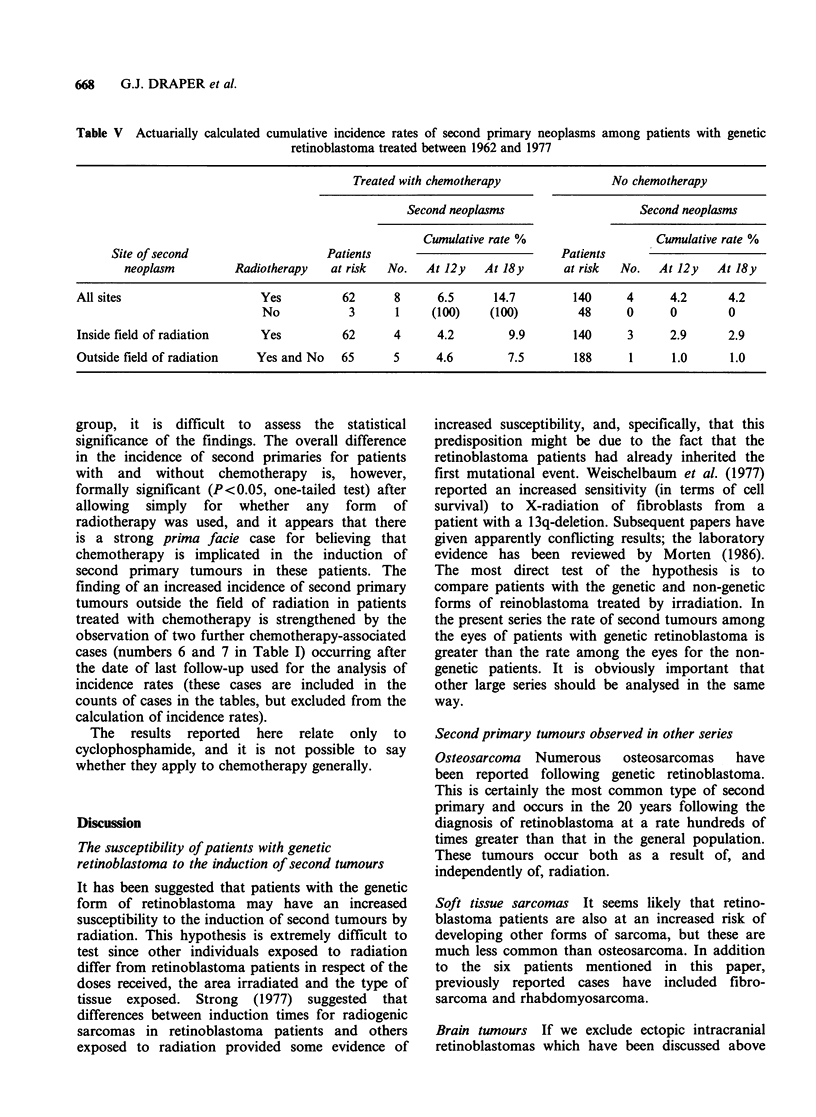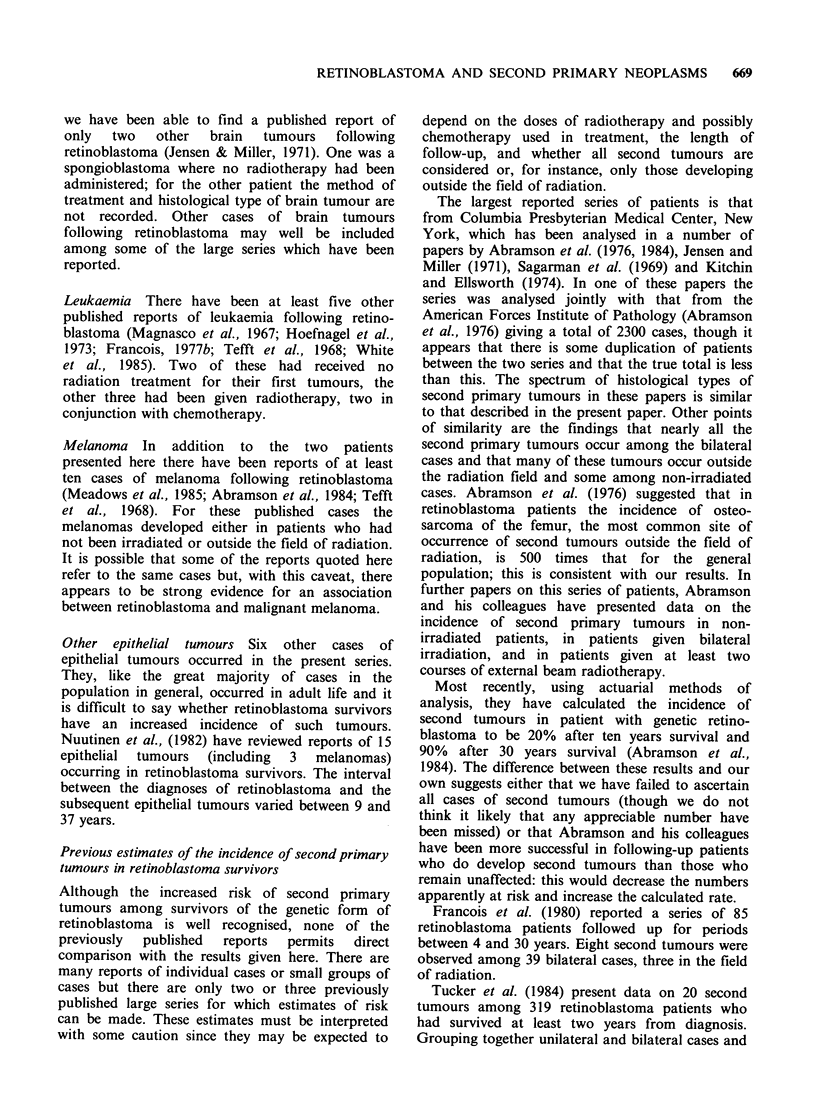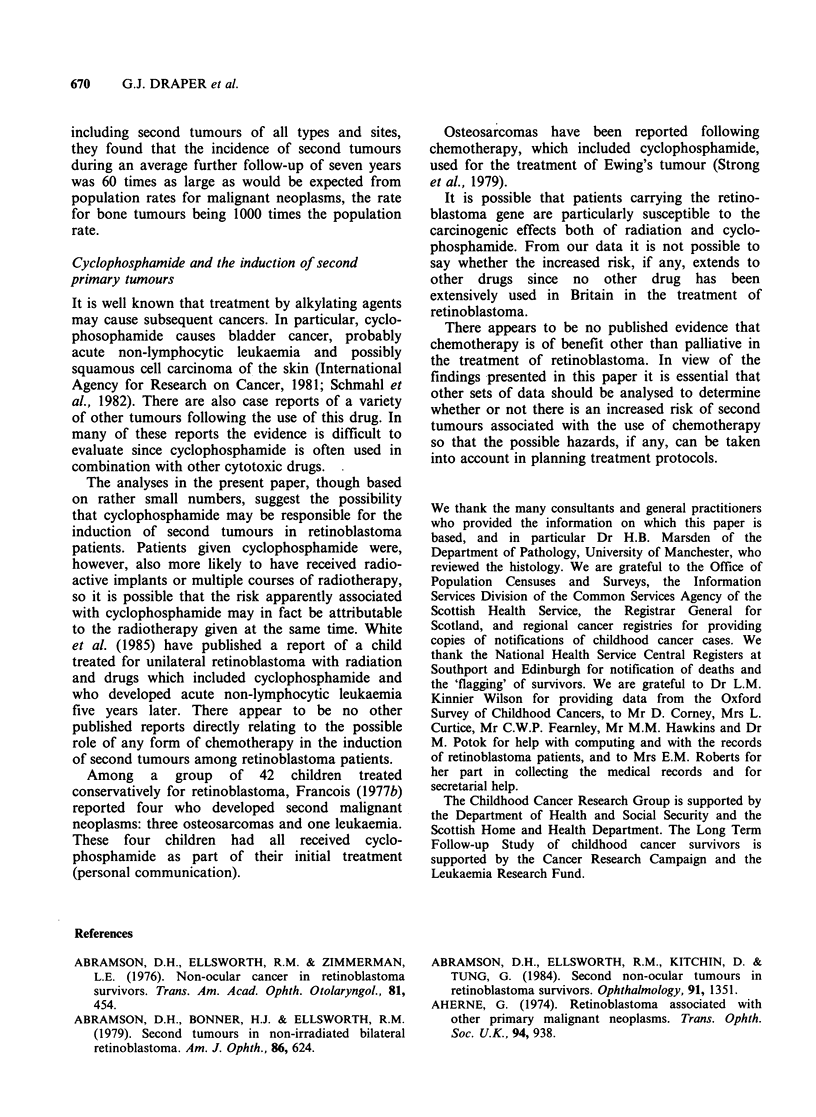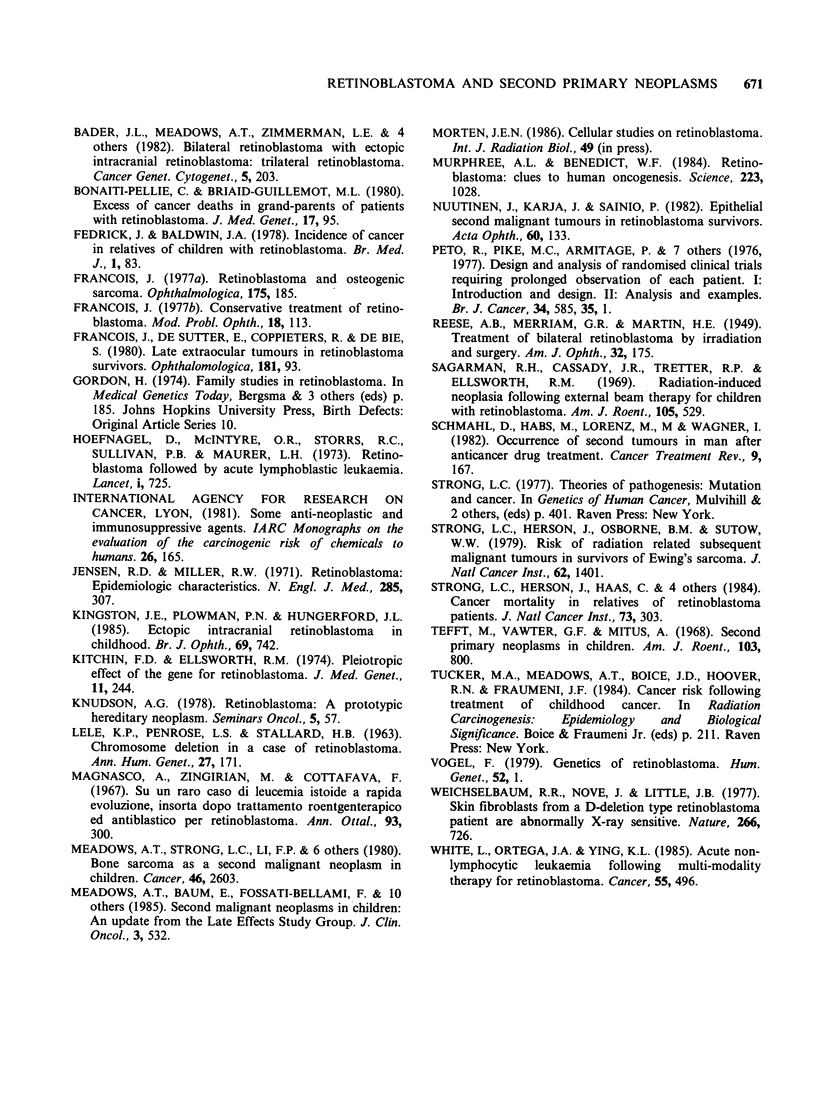# Second primary neoplasms in patients with retinoblastoma.

**DOI:** 10.1038/bjc.1986.110

**Published:** 1986-05

**Authors:** G. J. Draper, B. M. Sanders, J. E. Kingston

## Abstract

In a series of 882 retinoblastoma patients, 384 known to have the genetic form of the disease and 498 others, 30 patients developed second primary neoplasms. The spectrum of these second neoplasms is discussed in relation to the forms of treatment used for the retinoblastoma. Cumulative incidence rates of second tumours in the whole series are 2.0% at 12 years after diagnosis and 4.2% after 18 years. For patients with the genetic form of retinoblastoma the cumulative incidence rate after 18 years is 8.4% for all second neoplasms and 6.0% for osteosarcomas alone. The inherent risk among survivors from genetic retinoblastoma of developing an osteosarcoma, excluding all possible effects of treatment, is estimated to be 2.2% after 18 years. Within the field of radiation treatment the cumulative incidence rate for all second neoplasms after 18 years is 6.6% and for osteosarcomas alone 3.7%. There is some evidence that patients with genetic retinoblastoma are particularly sensitive to the carcinogenic effects of radiation. The results also suggest that the use of cyclophosphamide may increase the risk of second primary neoplasms in patients with genetic retinoblastoma. The incidence rates of second primary neoplasms in retinoblastoma survivors reported here are lower than those quoted for previously published series. Evidence from this and other papers strongly suggests an association between retinoblastoma and malignant melanoma.


*Br. J. Cancer* (1986), **53**, 661–671

### Second primary neoplasms in patients with retinoblastoma

G.J. Draper[1], B.M. Sanders[1] & J.E. Kingston[2]

[1]*Childhood Cancer Research Group, Department of Paediatrics, University of Oxford and* [2]*Department of Paediatric Oncology, St. Bartholomew's Hospital, London, UK.*

**Summary** In a series of 882 retinoblastoma patients, 384 known to have the genetic form of the disease and 498 others, 30 patients developed second primary neoplasms. The spectrum of these second neoplasms is discussed in relation to the forms of treatment used for the retinoblastoma. Cumulative incidence rates of second tumours in the whole series are 2.0% at 12 years after diagnosis and 4.2% after 18 years. For patients with the genetic form of retinoblastoma the cumulative incidence rate after 18 years is 8.4% for all second neoplasms and 6.0% for osteosarcomas alone. The inherent risk among survivors from genetic retinoblastoma of developing an osteosarcoma, excluding all possible effects of treatment, is estimated to be 2.2% after 18 years. Within the field of radiation treatment the cumulative incidence rate for all second neoplasms after 18 years is 6.6% and for osteosarcomas alone 3.7%. There is some evidence that patients with genetic retinoblastoma are particularly sensitive to the carcinogenic effects of radiation. The results also suggest that the use of cyclophosphamide may increase the risk of second primary neoplasms in patients with genetic retinoblastoma. The incidence rates of second primary neoplasms in retinoblastoma survivors reported here are lower than those quoted for previously published series. Evidence from this and other papers strongly suggests an association between retinoblastoma and malignant melanoma.

Retinoblastoma is a rare childhood tumour which can occur either unilaterally or bilaterally, and which may be either familial or sporadic. Cases known to be familial, and those with bilateral disease, usually show a pattern of inheritance consistent with the view that the occurrence of the tumour is attributable to a dominant autosomal gene with a penetrance of around 90%. We shall refer to familial and bilateral cases as 'genetic'.

Patients who survive genetic retinoblastoma have a substantial risk of developing osteosarcoma, probably soft tissue sarcomas and possibly other types of neoplasm. Reese *et al.* (1949) reported two second tumours following treatment of 55 cases of bilateral retinoblastoma with combined radiation and surgery, and the high risk of second malignant neoplasms in retinoblastoma survivors has subsequently been well documented (Aherne, 1974; Abramson *et al.*, 1976; Meadows *et al.*, 1980; 1985). These second primary neoplasms can often be attributed to radiotherapy but many cases have been reported of second tumours occurring outside the field of radiation treatment (Aherne, 1974; Abramson *et al.*, 1976), and, in some instances, where no radiotherapy had been given (Abramson *et al.*, 1979). It has been suggested that patients with genetic retinoblastoma are particularly susceptible to the carcinogenic effects of radiation, but there appears to be no direct quantitative assessment of this apparent susceptibility. Indeed little information is available on the actual magnitude of the risk of second primary tumours among retinoblastoma survivors.

Patients with the non-genetic form of the disease do not appear to have a particularly high risk of second neoplasms.

The association of other cancers with genetic retinoblastoma can be manifested in ways other than the increased risk to the survivors of developing a second tumour. These may include an increased risk of osteosarcoma in apparently unaffected gene carriers (Gordon, 1974; Francois, 1977*a*), and a generally increased risk of cancer in relatives of patients with retinoblastoma (Gordon, 1974; Bonaiti-Pellie & Briaid-Guillemot, 1980; Fedrick & Baldwin, 1978; Strong *et al.*, 1984).

The development of ectopic intracranial retinoblastoma, thought possibly to arise in cells of photoreceptor origin outside the retina, has been reported by Bader *et al.* (1982) and has been described as 'trilateral retinoblastoma'.

A chromosomal deletion associated with retinoblastoma was originally reported by Lele *et al.* (1963). It has since been shown that such cases involve a deletion located on band q14 of chromosome 13 and are sometimes associated with congenital abnormality and mental defect. Published cases were reviewed by Vogel (1979) and many further reports have since appeared.

Knudson (1978) suggested that retinoblastoma occurs as a result of two mutational events in homologous genes on each of a pair of chromosomes (i.e. that at the cell level it is a recessive condition) and that in the genetic form of the disease the first of these mutations is inherited. He also suggested that the mutations leading to

Correspondence: G.J. Draper
Received 16 July 1985; and in final form, 2 January 1986

© The Macmillan Press Ltd., 1986

retinoblastoma involved chromosome 13, and might create an 'immortal' cell line which continued to divide rather than differentiating. Murphree and Benedict (1984) suggested that the dominant allele of the retinoblastoma gene is a 'suppressor' or regulating gene, in the absence of which a retinoblastoma can occur, and considered possible mechanisms by which homozygosity or hemizygosity of the recessive allele might arise at the cell level.

In this paper we analyse the incidence rate of second primary tumours among retinoblastoma patients treated in Britain mainly between 1950 and 1977, particularly in relation to treatment and to whether the patient had the genetic or non-genetic form of the disease. We have also used these data together with those from some additional patients with double primary tumours to study the patterns of occurrence of second tumours and to draw a number of more qualitative conclusions.

### Materials and methods

The analysis presented here is based on records of children with retinoblastoma obtained from cancer registries and hospitals in England, Scotland and Wales. For the years 1962–1977 all cases notified to the National Cancer Registration Scheme have been included. For earlier years we have included only three-year survivors from retinoblastoma ascertained at certain hospitals and cancer registries. We shall refer to the 882 patients in these two groups as the 'follow-up' series.

Information about the age of the child at diagnosis of the retinoblastoma, whether the tumour was unilateral or bilateral, whether there was any known family history, methods of treatment and other details were obtained through cancer registries, hospitals and general practitioners.

Follow-up information was obtained both by writing to general practitioners and hospitals and through national record systems. In this way an 'effective follow-up' date was defined for each patient; we are confident that all or virtually all of the deaths or second primary tumours occurring before this date will have been identified. The effective follow-up date, or date of second primary tumour or death, if earlier, has been used as a 'cut-off' or 'censoring' date in the analyses described below.

By these methods a total of 30 second primary tumours were ascertained.

Nine further cases of retinoblastoma followed by a second primary tumour, but not included in the follow-up series, have been ascertained in other ways: among children who were included in a national survey of childhood cancer deaths there were three who died of osteosarcoma having previously been treated for retinoblastoma; a further six patients were ascertained through the records of their children who had themselves also had retinoblastoma.

For each of the double primary tumours the histological reports, or, wherever possible, the original slides, have been reviewed.

We have classified children with bilateral retinoblastoma and those for whom there is a known family history as 'genetic' cases and the remainder as 'non-genetic'. It is to be expected that some of those classified as non-genetic will in fact be unilateral cases with genetic retinoblastoma who have been wrongly classified because we have no record of a family history.

The treatment details recorded included the type of radiotherapy used and, for external beam radiotherapy, the tumour dose for each eye. For patients given chemotherapy the names of all drugs used were also recorded. Chemotherapy was sometimes given as palliative treatment for patients with metastases or whose retinoblastoma was at an advanced stage. In this series there were no survivors after extra-ocular recurrence of retinoblastoma.

### Results

Table I gives information about the 30 retinoblastoma survivors in the follow-up series who were discovered to have developed a second tumour. This table and the subsequent analyses of second primary tumours include all malignant neoplasms and all neoplasms of the brain and CNS occurring among the survivors, except for certain tumours of the pineal and suprasellar regions – so-called trilateral retinoblastoma. Table II shows the nine other retinoblastoma survivors developing second tumours but not included in the follow-up series.

A total of 268 patients were identified before 1962 of whom 131 (49%) had the genetic form of retinoblastoma, and 614 were identified from 1962 to 1977 of whom 253 (41%) had the genetic form of the disease. The rather high proportion of genetic cases in the earlier period is accounted for by the fact that some of these cases were identified through centres specialising in the treatment of retinoblastoma. The numbers of patients in each category and the forms of treatment used, together with the numbers of second tumours, are set out in Table III.

Six further patients who survived their initial treatment for retinoblastoma developed tumours in the pineal and suprasellar regions, after intervals of between two and six years. All of these children had retinoblastoma in both eyes. The histology of these 'trilateral' tumours is indistinguishable from retinoblastoma; the tumours have therefore been regarded as similar to bilateral retinoblastoma and have not been included in our series of second primary tumours. They are included in a paper on ectopic intracranial retinoblastoma (Kingston *et al.*, 1985).

*Second tumour type and possible association with treatment*

*Osteosarcoma* It is well known that this is the most common type of second tumour following retinoblastoma. In the present series, osteosarcomas developed in 21 retinoblastoma survivors: eight were sited within the field of radiation and three patients had additionally been given cyclophosphamide. The other 13 patients all developed osteosarcomas in the leg and five had been given cyclophosphamide.

*Soft tissue sarcomas* Six patients developed second tumours of this type, only two could be attributed to radiotherapy given for retinoblastoma. One patient was also treated with cyclophosphamide.

*Brain tumours* All three patients who developed second neoplasms in the brain had been treated with radiation, one had also been given cyclophosphamide.

*Leukaemia* One patient had received radiotherapy to both orbits and developed acute lymphatic leukaemia within three years of his initial tumour.

*Melanoma* One patient developed a melanoma within the radiation field seventeen years after treatment for retinoblastoma; the second patient whose melanoma developed after an interval of nearly 30 years had not received radiotherapy.

*Other epithelial tumours* These tumours developed after a much longer interval than most of the other second neoplasms; none of them could be attributed to radiation treatment. One patient developed three separate neoplasms after retinoblastoma; only the first of these tumours, an adenocarcinoma of the cervix, has been included in the analysis.

In the total of 39 patients included in Tables I and II, 27 received radiation treatment for their retinoblastoma. Fifteen of the second tumours developed within the field of this radiation and it is assumed that radiation is involved in their causation. Cyclophosphamide was included in the treatment of ten of the patients: the possible importance of this as a carcinogenic factor is discussed later.

*Calculation of incidence rates of second primary tumours*

In this and the following sections we give the results of analyses of actuarially calculated cumulative incidence rates of second tumours for the 882 patients in the follow-up series. The method used, which allows for varying lengths of follow-up for the patients included in the series, is that of Peto *et al.* (1976, 1977), normally used for the analysis of survival data: in the present context 'survival' is interpreted as survival free of a second tumour.

For all cases taken together the incidence of second primary tumours is estimated to be 2.0% at 12 years after diagnosis and 4.2% after 18 years. However such overall rates do not mean very much, and cannot, for instance, be used to compare different series because they depend on the proportions of genetic and non-genetic cases and on the number of patients given radiotherapy. These, and possibly other factors, will affect the chance that a second tumour will develop.

*Second primary tumours occurring among the genetic cases in the follow-up series*

Most of the second tumours in the follow-up series, 26 out of 30 cases, occurred among the 384 genetic cases, 25 in patients with bilateral tumours and the remaining one in a patient with a known family history of retinoblastoma. Actuarially calculated cumulative incidence rates at 12 and 18 years after the initial diagnosis are presented in Table IV. (Cases 6 and 7 in Table I are included in Table IV but for these patients the second tumours occurred after the date which defined the period of follow-up and they are therefore excluded from all analyses of incidence rates.) At 18 years after diagnosis the estimated rate of second primary tumours is 8.4%; for osteosarcomas alone the corresponding rate is 6.0%.

*Tumours outside the radiation field* As shown in Table IV, 12 of the 26 second primaries among the cases of genetic retinoblastoma developed outside the radiation field. Of these tumours, nine were osteosarcomas, two were soft-tissue sarcomas and one an epithelial tumour. The estimated cumulative incidence rate 18 years after the diagnosis of retinoblastoma is 3.0%, all of the second tumours observed within this period being osteosarcomas.

An estimate of the inherent risk of osteosarcoma among patients with genetic retinoblastoma can be

**Table I** Second primary neoplasms in patients in 'follow-up series'

| | *Retinoblastoma* | | | | | | | *Second Primary neoplasm* | |
|---|---|---|---|---|---|---|---|---|---|
| | | | | | *Treatment* | | | | |
| *Case no.* | *Side* | *Family history* | *Year of diagnosis* | *Age at diagnosis (months)* | *Enucleation* | *Radiotherapy (tumour dose in Gy)* | *Chemotherapy* | *Interval since retinoblastoma (months)* | *Diagnosis* |
| 1 | Bilateral | No | 1950 | 12 | R | L Radon seeds<br>R orbit radon seeds | Nil | 150 | Osteosarcoma R tibia |
| 2 | Left | No | 1951 | 4 | L | Nil | Nil | 155 | Osteosarcoma L fibula |
| 3 | Bilateral | Yes | 1958 | 3 | R L | L Radiotherapy | Nil | 170 | Osteosarcoma R femur |
| 4 | Bilateral | Yes | 1961 | 5 | R L | Nil | Nil | 142 | Osteosarcoma L tibia |
| 5 | Bilateral | No | 1963 | 19 | L | R Cobalt plaque ×2 | Cyclophosphamide | 187 | Osteosarcoma R tibia |
| 6 | Bilateral | No | 1965 | 18 | R L | L 35 Gy | Cyclophosphamide | 220 | Osteosarcoma R femur |
| 7 | Bilateral | Yes | 1968 | 6 | — | R 35 Gy<br>L 35 Gy | Cyclophosphamide | 184 | Osteosarcoma R femur |
| 8 | Bilateral | Yes | 1969 | 26 | R | L 62 Gy | Cyclophosphamide | 137 | Osteosarcoma L femur |
| 9 | Bilateral | No | 1970 | 12 | R | L 40 Gy | Nil | 119 | Osteosarcoma L femur |
| 10 | Right | Yes | 1971 | 3 | — | Nil | Cyclophosphamide | 120 | Osteosarcoma L tibia |
| 11 | Bilateral | No | 1948 | 8 | R L | R Radium plaque<br>R 12 Gy | Nil | 160 | Osteosarcoma R orbit |
| 12 | Bilateral | No | 1949 | 4 | R L | L Radon seeds L 44 Gy | Nil | 111 | Osteosarcoma L orbit |
| 13 | Bilateral | Yes | 1951 | 14 | R L | R 35 Gy | Nil | 96 | Osteosarcoma R orbit |
| 14 | Bilateral | No | 1960 | 17 | R L | R Cobalt plaque ×5 | Cyclophosphamide | 124 | Osteosarcoma R maxilla |
| 15 | Bilateral | No | 1961 | 28 | R L | R 50 Gy | Nil | 198 | Osteosarcoma nasal bones |
| 16 | Bilateral | No | 1966 | 16 | R L | R 80 Gy R iridium ring | Cyclophosphamide and TEM | 69 | Osteosarcoma R orbit |
| 17 | Bilateral | Yes | 1966 | 4 | — | R 35 Gy<br>R L Cobalt plaques | Cyclophosphamide | 105 | Osteosarcoma R orbit |
| 18 | Bilateral | Yes | 1972 | 4 | L | R 39 Gy<br>L 55 Gy | Nil | 59 | Osteosarcoma L orbit |
| 19 | Bilateral | No | 1964 | 18 | R L | R 40 Gy R Cobalt plaque ×2 | Cyclophosphamide | 167 | Fibrosarcoma R orbit |
| 20 | Bilateral | Yes | 1966 | 15 | R L | R Cobalt plaque ×3 | Nil | 113 | Anaplastic fibrosarcoma R orbit |
| 21 | Bilateral | Yes | 1974 | 27 | R L | R 35 Gy<br>L orbit 45 Gy | Nil | 31 | Acute lymphatic leukaemia |
| 22 | Bilateral | No | 1964 | 6 | R | L 30 Gy L Cobalt plaque | Cyclophosphamide | 187 | Glioblastoma L temporal lobe |

| | | | | | | | | | |
|---|---|---|---|---|---|---|---|---|---|
| 23 | Bilateral | No | 1946 | 20 | L | L 15 Gy<br>R Radium implant | Nil | 409 | Astrocytoma Suprasellar |
| 24 | Left | No | 1945 | 17 | L | Cranium 30 Gy | Nil | 375 | Meningioma (benign) |
| 25 | Bilateral | Yes | 1954 | 7 | R | L Cobalt plaque | Nil | 210 | Malignant melanoma L forehead |
| 26 | Bilateral | No | 1927 | 11 | R L | Nil | Nil | 625<br>635<br>637 | Adenocarcinoma cervix<br>Leiomyosarcoma R temple<br>Sebaceous carcinoma eyelid |
| 27 | Right | No | 1935 | 11 | R | Nil | Nil | 531 | Carcinoma oesophagus |
| 28 | Left | No | 1949 | 25 | L | L Radon seeds | Nil | 346 | Carcinoma L breast |
| 29 | Bilateral | Yes | 1947 | 1 | R | L Radon seeds | Nil | 363 | Liposarcoma (retroperitoneal) |
| 30 | Bilateral | Yes | 1952 | 6 | R L | R Radon seeds<br>R Tantalum implant | Nil | 310 | Sarcoma stomach |

**Table II** Second primary neoplasms in patients not in 'follow-up series'

| | *Retinoblastoma* | | | | | | | *Second Primary neoplasm* | |
|---|---|---|---|---|---|---|---|---|---|
| | | | | | *Treatment* | | | | |
| *Case no.* | *Side* | *Family history* | *Year of diagnosis* | *Age at diagnosis (months)* | *Enucleation* | *Radiotherapy (tumour dose in Gy)* | *Chemotherapy* | *Interval since retinoblastoma (months)* | *Diagnosis* |
| 31 | Left | No | 1952 | 24 | L | Nil | Nil | 140 | Osteosarcoma L femur |
| 32 | Bilateral | No | 1954 | 27 | L | R Radon seeds | Nil | 144 | Osteosarcoma L femur |
| 33 | Right | Yes | 1964 | 15 | R | Nil | Nil | 132 | Osteosarcoma L femur |
| 34 | Left | Yes | 1934 | 14 | L | Nil | Nil | 226 | Fibrosarcoma L femur |
| 35 | Bilateral | Yes | 1945 | 34 | R L | Nil | Nil | 380 | Adenocarcinoma bronchus |
| 36 | Bilateral | Yes | 1941 | 36 | L | R Radon seeds | Nil | 480 | Anaplastic carcinoma R orbit or lung |
| 37 | Bilateral | Yes | 1924 | NK | NK | NK | NK | 360 | Granulosa cell tumour ovary |
| 38 | Bilateral | Yes | 1946 | 15 | R L | Nil | Nil | 340 | Malignant melanoma L foot |
| 39 | Bilateral | Yes | 1920 | 18 | R L | Nil | Nil | 549 | Spindle cell sarcoma (Primary site NK) |

**Table III** Second primary neoplasms occurring among patients in 'follow-up series' according to type of retinoblastoma, year of diagnosis and type of treatment (second tumours/patients)

| Type of retinoblastoma | Year of diagnosis | Radiotherapy: Chemotherapy | Radiotherapy: No chemotherapy | Radiotherapy: All | No radiotherapy: Chemotherapy | No radiotherapy: No chemotherapy | No radiotherapy: All |
|---|---|---|---|---|---|---|---|
| Genetic | Pre 1962 | 1/11 | 10/101 | 11/112 | 0/0 | 2/19 | 2/19 |
| | 1962–77 | 8/62 | 4/140 | 12/202 | 1/3 | 0/48 | 1/51 |
| | All years | 9/73 | 14/241 | 23/314 | 1/3 | 2/67 | 3/70 |
| Non-genetic | Pre-1962 | 0/1 | 2/24 | 2/25 | 0/0 | 2/112 | 2/112 |
| | 1962–77 | 0/21 | 0/54 | 0/75 | 0/8 | 0/278 | 0/286 |
| | All years | 0/22 | 2/78 | 2/100 | 0/8 | 2/390 | 2/398 |
| Total | All years | 9/95 | 16/319 | 25/414 | 1/11 | 4/457 | 5/468 |

**Table IV** Actuarially calculated cumulative incidence rates of second primary neoplasms among patients with genetic retinoblastoma

| Site of second neoplasm | Treatment group | Patients at risk | All second neoplasms: No. | Cumulative rate %: At 12 y | Cumulative rate %: At 18 y | Osteosarcoma: No. | Cumulative rate %: At 12 y | Cumulative rate %: At 18 y |
|---|---|---|---|---|---|---|---|---|
| All sites | All | 384 | 26 | 4.3 | 8.4 | 17 | 3.6 | 6.0 |
| Inside field of radiation | Radiotherapy | 314 | 14 | 3.4 | 6.6 | 8 | 2.4 | 3.7 |
| Outside field of radiation | All | 384 | 12 | 1.6 | 3.0 | 9 | 1.6 | 3.0 |
| (includes non-irradiated) | No chemotherapy | 308 | 7 | 1.0 | 2.2 | 4 | 1.0 | 2.2 |

obtained by considering only the osteosarcomas occurring outside the radiation field, and excluding all patients who received chemotherapy. There were 308 such patients and in four of them an osteosarcoma developed in a leg-bone. This gives an actuarially estimated rate of 2.2% by 18 years. For the population in general about one person in 10,000 would develop an osteosarcoma during a similar period. (The majority of such tumours occur in the long bones of the leg.) The risk for patients with the genetic form of retinoblastoma, excluding any risk from treatment, is therefore estimated to be about 200 times that for the general population. Since this ratio is based on only four cases, the estimate is very imprecise; 95% confidence limits are approximately 50 and 500.

*Tumours within the radiation field* Table IV also shows the incidence rate for tumours within the radiation field for genetic cases receiving radiotherapy. We have, as explained above, included in this category the two brain tumours, the melanoma of the forehead, and the leukaemia, as well as the osteosarcomas and fibrosarcomas of the orbit, maxilla and nasal bones. These 14 tumours give an estimated incidence rate by 18 years of 6.6%. The corresponding rate for osteosarcomas alone is 3.7%. Osteosarcomas of the orbit are very rare, and probably less than 10% of all osteosarcomas occur at any site in the skull and jaw bones in this age-group; we would expect that less than one person in 100,000 would develop such a tumour in the first 20 years of life. The observed rate is of the order of 4000 times as great as this.

### *Comparisons of incidence rates between various sub-groups of cases*

In the next two sections we compare the incidence rates of second primary neoplasms between patients with genetic and non-genetic retinoblastoma and then between patients given or not given chemotherapy. In most cases we have not been able to give statistical significance levels, first because some of the rates are calculated in a non-standard way and secondly because the small numbers of second tumours observed mean that the usual approximations are not valid here. Also the number of overlapping analyses carried out and the differences in radiotherapy regimes for the sub-groups of cases of interest vitiate such analyses. The conclusions suggested here must therefore be regarded as tentative and awaiting confirmation. We believe, nevertheless, that they are reasonable in the light of other knowledge about retinoblastoma.

*Comparisons between the incidence of second primary tumours in patients with the genetic and non-genetic forms of retinoblastoma*

Among the 498 patients classified, on the evidence available to us, as having the non-genetic form of retinoblastoma only four second primary tumours were recorded, three outside and one inside the field of radiation. Only one of these four tumours, an osteosarcoma of the fibula, occurred in the first 18 years following the diagnosis of retinoblastoma; it seems probable that this patient did in fact have the genetic form of the disease. The incidence rate for second primary tumours outside the field of radiation, based on this one case is 0.4% at 18 years; if this case was indeed wrongly classified the rate is zero. The corresponding rate for the genetic cases is 3.0%.

For tumours inside the field of radiation the contrast between the rates for genetic and non-genetic cases is also striking: the only such tumour in the 100 patients with non-genetic retinoblastoma given radiotherapy, a meningioma of the brain, occurred 31 years after treatment; the estimated rates for the patients with genetic retinoblastoma are 3.4% at 12 years and 6.6% at 18 years.

Ideally the analysis of radiation-induced second tumours should take account of the dose and type of radiotherapy, and in a subsequent study we propose to carry out such an analysis. In the treatment of patients reported here several different forms of implant as well as external beam radiotherapy were used; for the latter the doses ranged from 12 to 75 Gy, though the majority were in the range 35–40 Gy. For the present paper we have simply re-analysed the data on the occurrence of second primaries in the field of radiation taking into account the number of eyes irradiated rather than the number of patients, and calculating tumour rates per 100 eyes irradiated. For patients with genetic retinoblastoma a total of 380 eyes were irradiated and 14 tumours were observed within the field of radiation; the cumulative rates of occurrence of such tumours are 2.9% after 12 years and 5.7% after 18 years. These rates are less than those quoted for the incidence rates in terms of numbers of patients because the number of eyes irradiated is greater than the number of patients. For the 100 patients with non-genetic retinoblastoma (always unilateral) no second primaries were observed within the field of radiation in the 18 years after diagnosis. These results suggest that patients with the genetic form of retinoblastoma may be more susceptible to the induction of second tumours by radiation, though we have not taken into account differences in radiation doses between genetic and non-genetic cases.

The effects of chemotherapy are discussed in the next section. Chemotherapy was used for a greater proportion of genetic than of non-genetic cases but this does not appear to account for the higher incidence of second primaries in the genetic cases.

*Effect of chemotherapy*

Ninety patients received cyclophosphamide as part of their initial treatment for retinoblastoma, sometimes in combination with other drugs. Ten of these patients developed second primary tumours, eight osteosarcomas, one fibrosarcoma and one glioblastoma. Sixteen patients received other forms of chemotherapy and none of these developed a second tumour.

The majority of the patients who received chemotherapy were genetic cases who also received radiotherapy; chemotherapy was seldom used before 1962 and our records for earlier years are incomplete. We have therefore restricted the detailed analysis of the possible risks associated with chemotherapy to patients with the genetic form of retinoblastoma treated from 1962 onwards.

Table V shows the second neoplasm rates for patients with genetic retinoblastoma treated in the years 1962–1977: the patients treated with chemotherapy are compared with those who received none with respect to (a) all neoplasms occurring among patients given radiotherapy, (b) all neoplasms among those not given radiotherapy, (c) neoplasms in the radiation field, and (d) neoplasms outside the radiation field. For each of these comparisons the incidence of second primary tumours is greater among patients given chemotherapy than among those not given it, though of course the second neoplasms in (a) and (b) are the same as those in (c) and (d). The estimated incidence rates 12 years after diagnosis for tumours inside the field of radiation are 4.2% for patients given chemotherapy in addition to radiation and 2.9% for patients not given chemotherapy. The rates for tumours outside the field of radiation (and including also patients who were not irradiated at all) are 4.6% and 1.0% respectively for patients with and without chemotherapy. At 18 years after diagnosis the contrasts are even more striking. For tumours inside the field an analysis taking into account the numbers of eyes irradiated yields similar results. For reasons given above, and in particular the fact that patients who received chemotherapy were also more likely to have had repeated courses of radiotherapy which might have been responsible for the higher incidence of second primaries in this

**Table V** Actuarially calculated cumulative incidence rates of second primary neoplasms among patients with genetic retinoblastoma treated between 1962 and 1977

| | | *Treated with chemotherapy* | | | | *No chemotherapy* | | | |
|---|---|---|---|---|---|---|---|---|---|
| | | | *Second neoplasms* | | | | *Second neoplasms* | | |
| | | | | *Cumulative rate %* | | | | *Cumulative rate %* | |
| *Site of second neoplasm* | *Radiotherapy* | *Patients at risk* | *No.* | *At 12 y* | *At 18 y* | *Patients at risk* | *No.* | *At 12 y* | *At 18 y* |
| All sites | Yes | 62 | 8 | 6.5 | 14.7 | 140 | 4 | 4.2 | 4.2 |
| | No | 3 | 1 | (100) | (100) | 48 | 0 | 0 | 0 |
| Inside field of radiation | Yes | 62 | 4 | 4.2 | 9.9 | 140 | 3 | 2.9 | 2.9 |
| Outside field of radiation | Yes and No | 65 | 5 | 4.6 | 7.5 | 188 | 1 | 1.0 | 1.0 |

group, it is difficult to assess the statistical significance of the findings. The overall difference in the incidence of second primaries for patients with and without chemotherapy is, however, formally significant ($P < 0.05$, one-tailed test) after allowing simply for whether any form of radiotherapy was used, and it appears that there is a strong *prima facie* case for believing that chemotherapy is implicated in the induction of second primary tumours in these patients. The finding of an increased incidence of second primary tumours outside the field of radiation in patients treated with chemotherapy is strengthened by the observation of two further chemotherapy-associated cases (numbers 6 and 7 in Table I) occurring after the date of last follow-up used for the analysis of incidence rates (these cases are included in the counts of cases in the tables, but excluded from the calculation of incidence rates).

The results reported here relate only to cyclophosphamide, and it is not possible to say whether they apply to chemotherapy generally.

### Discussion

### *The susceptibility of patients with genetic retinoblastoma to the induction of second tumours*

It has been suggested that patients with the genetic form of retinoblastoma may have an increased susceptibility to the induction of second tumours by radiation. This hypothesis is extremely difficult to test since other individuals exposed to radiation differ from retinoblastoma patients in respect of the doses received, the area irradiated and the type of tissue exposed. Strong (1977) suggested that differences between induction times for radiogenic sarcomas in retinoblastoma patients and others exposed to radiation provided some evidence of increased susceptibility, and, specifically, that this predisposition might be due to the fact that the retinoblastoma patients had already inherited the first mutational event. Weischelbaum *et al.* (1977) reported an increased sensitivity (in terms of cell survival) to X-radiation of fibroblasts from a patient with a 13q-deletion. Subsequent papers have given apparently conflicting results; the laboratory evidence has been reviewed by Morten (1986). The most direct test of the hypothesis is to compare patients with the genetic and non-genetic forms of reinoblastoma treated by irradiation. In the present series the rate of second tumours among the eyes of patients with genetic retinoblastoma is greater than the rate among the eyes for the non-genetic patients. It is obviously important that other large series should be analysed in the same way.

### *Second primary tumours observed in other series*

*Osteosarcoma* Numerous osteosarcomas have been reported following genetic retinoblastoma. This is certainly the most common type of second primary and occurs in the 20 years following the diagnosis of retinoblastoma at a rate hundreds of times greater than that in the general population. These tumours occur both as a result of, and independently of, radiation.

*Soft tissue sarcomas* It seems likely that retinoblastoma patients are also at an increased risk of developing other forms of sarcoma, but these are much less common than osteosarcoma. In addition to the six patients mentioned in this paper, previously reported cases have included fibrosarcoma and rhabdomyosarcoma.

*Brain tumours* If we exclude ectopic intracranial retinoblastomas which have been discussed above we have been able to find a published report of only two other brain tumours following retinoblastoma (Jensen & Miller, 1971). One was a spongioblastoma where no radiotherapy had been administered; for the other patient the method of treatment and histological type of brain tumour are not recorded. Other cases of brain tumours following retinoblastoma may well be included among some of the large series which have been reported.

*Leukaemia* There have been at least five other published reports of leukaemia following retinoblastoma (Magnasco *et al.*, 1967; Hoefnagel *et al.*, 1973; Francois, 1977*b*; Tefft *et al.*, 1968; White *et al.*, 1985). Two of these had received no radiation treatment for their first tumours, the other three had been given radiotherapy, two in conjunction with chemotherapy.

*Melanoma* In addition to the two patients presented here there have been reports of at least ten cases of melanoma following retinoblastoma (Meadows *et al.*, 1985; Abramson *et al.*, 1984; Tefft *et al.*, 1968). For these published cases the melanomas developed either in patients who had not been irradiated or outside the field of radiation. It is possible that some of the reports quoted here refer to the same cases but, with this caveat, there appears to be strong evidence for an association between retinoblastoma and malignant melanoma.

*Other epithelial tumours* Six other cases of epithelial tumours occurred in the present series. They, like the great majority of cases in the population in general, occurred in adult life and it is difficult to say whether retinoblastoma survivors have an increased incidence of such tumours. Nuutinen *et al.*, (1982) have reviewed reports of 15 epithelial tumours (including 3 melanomas) occurring in retinoblastoma survivors. The interval between the diagnoses of retinoblastoma and the subsequent epithelial tumours varied between 9 and 37 years.

*Previous estimates of the incidence of second primary tumours in retinoblastoma survivors*

Although the increased risk of second primary tumours among survivors of the genetic form of retinoblastoma is well recognised, none of the previously published reports permits direct comparison with the results given here. There are many reports of individual cases or small groups of cases but there are only two or three previously published large series for which estimates of risk can be made. These estimates must be interpreted with some caution since they may be expected to depend on the doses of radiotherapy and possibly chemotherapy used in treatment, the length of follow-up, and whether all second tumours are considered or, for instance, only those developing outside the field of radiation.

The largest reported series of patients is that from Columbia Presbyterian Medical Center, New York, which has been analysed in a number of papers by Abramson *et al.* (1976, 1984), Jensen and Miller (1971), Sagarman *et al.* (1969) and Kitchin and Ellsworth (1974). In one of these papers the series was analysed jointly with that from the American Forces Institute of Pathology (Abramson *et al.*, 1976) giving a total of 2300 cases, though it appears that there is some duplication of patients between the two series and that the true total is less than this. The spectrum of histological types of second primary tumours in these papers is similar to that described in the present paper. Other points of similarity are the findings that nearly all the second primary tumours occur among the bilateral cases and that many of these tumours occur outside the radiation field and some among non-irradiated cases. Abramson *et al.* (1976) suggested that in retinoblastoma patients the incidence of osteosarcoma of the femur, the most common site of occurrence of second tumours outside the field of radiation, is 500 times that for the general population; this is consistent with our results. In further papers on this series of patients, Abramson and his colleagues have presented data on the incidence of second primary tumours in non-irradiated patients, in patients given bilateral irradiation, and in patients given at least two courses of external beam radiotherapy.

Most recently, using actuarial methods of analysis, they have calculated the incidence of second tumours in patient with genetic retinoblastoma to be 20% after ten years survival and 90% after 30 years survival (Abramson *et al.*, 1984). The difference between these results and our own suggests either that we have failed to ascertain all cases of second tumours (though we do not think it likely that any appreciable number have been missed) or that Abramson and his colleagues have been more successful in following-up patients who do develop second tumours than those who remain unaffected: this would decrease the numbers apparently at risk and increase the calculated rate.

Francois *et al.* (1980) reported a series of 85 retinoblastoma patients followed up for periods between 4 and 30 years. Eight second tumours were observed among 39 bilateral cases, three in the field of radiation.

Tucker *et al.* (1984) present data on 20 second tumours among 319 retinoblastoma patients who had survived at least two years from diagnosis. Grouping together unilateral and bilateral cases and including second tumours of all types and sites, they found that the incidence of second tumours during an average further follow-up of seven years was 60 times as large as would be expected from population rates for malignant neoplasms, the rate for bone tumours being 1000 times the population rate.

*Cyclophosphamide and the induction of second primary tumours*

It is well known that treatment by alkylating agents may cause subsequent cancers. In particular, cyclophosophamide causes bladder cancer, probably acute non-lymphocytic leukaemia and possibly squamous cell carcinoma of the skin (International Agency for Research on Cancer, 1981; Schmahl *et al.*, 1982). There are also case reports of a variety of other tumours following the use of this drug. In many of these reports the evidence is difficult to evaluate since cyclophosphamide is often used in combination with other cytotoxic drugs.

The analyses in the present paper, though based on rather small numbers, suggest the possibility that cyclophosphamide may be responsible for the induction of second tumours in retinoblastoma patients. Patients given cyclophosphamide were, however, also more likely to have received radioactive implants or multiple courses of radiotherapy, so it is possible that the risk apparently associated with cyclophosphamide may in fact be attributable to the radiotherapy given at the same time. White *et al.* (1985) have published a report of a child treated for unilateral retinoblastoma with radiation and drugs which included cyclophosphamide and who developed acute non-lymphocytic leukaemia five years later. There appear to be no other published reports directly relating to the possible role of any form of chemotherapy in the induction of second tumours among retinoblastoma patients.

Among a group of 42 children treated conservatively for retinoblastoma, Francois (1977*b*) reported four who developed second malignant neoplasms: three osteosarcomas and one leukaemia. These four children had all received cyclophosphamide as part of their initial treatment (personal communication).

Osteosarcomas have been reported following chemotherapy, which included cyclophosphamide, used for the treatment of Ewing's tumour (Strong *et al.*, 1979).

It is possible that patients carrying the retinoblastoma gene are particularly susceptible to the carcinogenic effects both of radiation and cyclophosphamide. From our data it is not possible to say whether the increased risk, if any, extends to other drugs since no other drug has been extensively used in Britain in the treatment of retinoblastoma.

There appears to be no published evidence that chemotherapy is of benefit other than palliative in the treatment of retinoblastoma. In view of the findings presented in this paper it is essential that other sets of data should be analysed to determine whether or not there is an increased risk of second tumours associated with the use of chemotherapy so that the possible hazards, if any, can be taken into account in planning treatment protocols.

We thank the many consultants and general practitioners who provided the information on which this paper is based, and in particular Dr H.B. Marsden of the Department of Pathology, University of Manchester, who reviewed the histology. We are grateful to the Office of Population Censuses and Surveys, the Information Services Division of the Common Services Agency of the Scottish Health Service, the Registrar General for Scotland, and regional cancer registries for providing copies of notifications of childhood cancer cases. We thank the National Health Service Central Registers at Southport and Edinburgh for notification of deaths and the 'flagging' of survivors. We are grateful to Dr L.M. Kinnier Wilson for providing data from the Oxford Survey of Childhood Cancers, to Mr D. Corney, Mrs L. Curtice, Mr C.W.P. Fearnley, Mr M.M. Hawkins and Dr M. Potok for help with computing and with the records of retinoblastoma patients, and to Mrs E.M. Roberts for her part in collecting the medical records and for secretarial help.

The Childhood Cancer Research Group is supported by the Department of Health and Social Security and the Scottish Home and Health Department. The Long Term Follow-up Study of childhood cancer survivors is supported by the Cancer Research Campaign and the Leukaemia Research Fund.

### References

ABRAMSON, D.H., ELLSWORTH, R.M. & ZIMMERMAN, L.E. (1976). Non-ocular cancer in retinoblastoma survivors. *Trans. Am. Acad. Ophth. Otolaryngol.*, **81**, 454.

ABRAMSON, D.H., BONNER, H.J. & ELLSWORTH, R.M. (1979). Second tumours in non-irradiated bilateral retinoblastoma. *Am. J. Ophth.*, **86**, 624.

ABRAMSON, D.H., ELLSWORTH, R.M., KITCHIN, D. & TUNG, G. (1984). Second non-ocular tumours in retinoblastoma survivors. *Ophthalmology*, **91**, 1351.

AHERNE, G. (1974). Retinoblastoma associated with other primary malignant neoplasms. *Trans. Ophth. Soc. U.K.*, **94**, 938.

BADER, J.L., MEADOWS, A.T., ZIMMERMAN, L.E. & 4 others (1982). Bilateral retinoblastoma with ectopic intracranial retinoblastoma: trilateral retinoblastoma. *Cancer Genet. Cytogenet.*, **5**, 203.

BONAITI-PELLIE, C. & BRIAID-GUILLEMOT, M.L. (1980). Excess of cancer deaths in grand-parents of patients with retinoblastoma. *J. Med. Genet.*, **17**, 95.

FEDRICK, J. & BALDWIN, J.A. (1978). Incidence of cancer in relatives of children with retinoblastoma. *Br. Med. J.*, **1**, 83.

FRANCOIS, J. (1977*a*). Retinoblastoma and osteogenic sarcoma. *Ophthalmologica*, **175**, 185.

FRANCOIS, J. (1977*b*). Conservative treatment of retinoblastoma. *Mod. Probl. Ophth.*, **18**, 113.

FRANCOIS, J., DE SUTTER, E., COPPIETERS, R. & DE BIE, S. (1980). Late extraocular tumours in retinoblastoma survivors. *Ophthalomologica*, **181**, 93.

GORDON, H. (1974). Family studies in retinoblastoma. In *Medical Genetics Today*, Bergsma & 3 others (eds) p. 185. Johns Hopkins University Press, Birth Defects: Original Article Series 10.

HOEFNAGEL, D., McINTYRE, O.R., STORRS, R.C., SULLIVAN, P.B. & MAURER, L.H. (1973). Retinoblastoma followed by acute lymphoblastic leukaemia. *Lancet*, **i**, 725.

INTERNATIONAL AGENCY FOR RESEARCH ON CANCER, LYON, (1981). Some anti-neoplastic and immunosuppressive agents. *IARC Monographs on the evaluation of the carcinogenic risk of chemicals to humans*. **26**, 165.

JENSEN, R.D. & MILLER, R.W. (1971). Retinoblastoma: Epidemiologic characteristics. *N. Engl. J. Med.*, **285**, 307.

KINGSTON, J.E., PLOWMAN, P.N. & HUNGERFORD, J.L. (1985). Ectopic intracranial retinoblastoma in childhood. *Br. J. Ophth.*, **69**, 742.

KITCHIN, F.D. & ELLSWORTH, R.M. (1974). Pleiotropic effect of the gene for retinoblastoma. *J. Med. Genet.*, **11**, 244.

KNUDSON, A.G. (1978). Retinoblastoma: A prototypic hereditary neoplasm. *Seminars Oncol.*, **5**, 57.

LELE, K.P., PENROSE, L.S. & STALLARD, H.B. (1963). Chromosome deletion in a case of retinoblastoma. *Ann. Hum. Genet.*, **27**, 171.

MAGNASCO, A., ZINGIRIAN, M. & COTTAFAVA, F. (1967). Su un raro caso di leucemia istoide a rapida evoluzione, insorta dopo trattamento roentgenterapico ed antiblastico per retinoblastoma. *Ann. Ottal.*, **93**, 300.

MEADOWS, A.T., STRONG, L.C., LI, F.P. & 6 others (1980). Bone sarcoma as a second malignant neoplasm in children. *Cancer*, **46**, 2603.

MEADOWS, A.T., BAUM, E., FOSSATI-BELLAMI, F. & 10 others (1985). Second malignant neoplasms in children: An update from the Late Effects Study Group. *J. Clin. Oncol.*, **3**, 532.

MORTEN, J.E.N. (1986). Cellular studies on retinoblastoma. *Int. J. Radiation Biol.*, **49** (in press).

MURPHREE, A.L. & BENEDICT, W.F. (1984). Retinoblastoma: clues to human oncogenesis. *Science*, **223**, 1028.

NUUTINEN, J., KARJA, J. & SAINIO, P. (1982). Epithelial second malignant tumours in retinoblastoma survivors. *Acta Ophth.*, **60**, 133.

PETO, R., PIKE, M.C., ARMITAGE, P. & 7 others (1976, 1977). Design and analysis of randomised clinical trials requiring prolonged observation of each patient. I: Introduction and design. II: Analysis and examples. *Br. J. Cancer*, **34**, 585, **35**, 1.

REESE, A.B., MERRIAM, G.R. & MARTIN, H.E. (1949). Treatment of bilateral retinoblastoma by irradiation and surgery. *Am. J. Ophth.*, **32**, 175.

SAGARMAN, R.H., CASSADY, J.R., TRETTER, R.P. & ELLSWORTH, R.M. (1969). Radiation-induced neoplasia following external beam therapy for children with retinoblastoma. *Am. J. Roent.*, **105**, 529.

SCHMAHL, D., HABS, M., LORENZ, M., M & WAGNER, I. (1982). Occurrence of second tumours in man after anticancer drug treatment. *Cancer Treatment Rev.*, **9**, 167.

STRONG, L.C. (1977). Theories of pathogenesis: Mutation and cancer. In *Genetics of Human Cancer*, Mulvihill & 2 others, (eds) p. 401. Raven Press: New York.

STRONG, L.C., HERSON, J., OSBORNE, B.M. & SUTOW, W.W. (1979). Risk of radiation related subsequent malignant tumours in survivors of Ewing's sarcoma. *J. Natl Cancer Inst.*, **62**, 1401.

STRONG, L.C., HERSON, J., HAAS, C. & 4 others (1984). Cancer mortality in relatives of retinoblastoma patients. *J. Natl Cancer Inst.*, **73**, 303.

TEFFT, M., VAWTER, G.F. & MITUS, A. (1968). Second primary neoplasms in children. *Am. J. Roent.*, **103**, 800.

TUCKER, M.A., MEADOWS, A.T., BOICE, J.D., HOOVER, R.N. & FRAUMENI, J.F. (1984). Cancer risk following treatment of childhood cancer. In *Radiation Carcinogenesis: Epidemiology and Biological Significance*. Boice & Fraumeni Jr. (eds) p. 211. Raven Press: New York.

VOGEL, F. (1979). Genetics of retinoblastoma. *Hum. Genet.*, **52**, 1.

WEICHSELBAUM, R.R., NOVE, J. & LITTLE, J.B. (1977). Skin fibroblasts from a D-deletion type retinoblastoma patient are abnormally X-ray sensitive. *Nature*, **266**, 726.

WHITE, L., ORTEGA, J.A. & YING, K.L. (1985). Acute non-lymphocytic leukaemia following multi-modality therapy for retinoblastoma. *Cancer*, **55**, 496.